# Symmetrical dental occlusion blocking – changes of body sway and weight distribution in healthy subjects across 4 age decades

**DOI:** 10.1186/s12995-021-00296-1

**Published:** 2021-02-27

**Authors:** C. Maurer-Grubinger, F. Adjami, I. Avaniadi, W. Christian, C. Doerry, V. Fay, V. Fisch, A. Gerez, J. Goecke, U. Kaya, J. Keller, D. Krüger, J. Pflaum, L. Porsch, C. Wischnewski, B. Scharnweber, P. Sosnov, G. Oremek, D. A. Groneberg, D. Ohlendorf

**Affiliations:** 1 Institute of Occupational Medicine, Social Medicine and Environmental Medicine, Goethe-University Frankfurt/Main, Theodor-Stern-Kai 7, Building 9A, 60590 Frankfurt/Main, Germany; 2 Department of Orthodontics, School of Dentistry “Carolinum”, Goethe-University Frankfurt am Main, Theodor-Stern-Kai 7, Building 29, 60590 Frankfurt/Main, Germany

**Keywords:** Meersseman test, Healthy adults, Weight distribution, Symmetrical blocked occlusion, Body sway

## Abstract

**Objectives:**

Symmetrical dental occlusion blocking is used in dentistry as a quick diagnostic tool to test for potential influences of the craniomandibular system on body sway and weight distribution. This study presents the changes of body sway and pressure distribution in healthy subjects, free of a temporomandibular dysfunction (TMD). Immediate effects between occlusal blocking and rest position on body sway and body weight distribution in general, as well as for both genders and for four age decades will be evaluated.

**Materials and methods:**

725 (396f/329 m) subjects (neither subjective signs of TMD nor acute/chronic complaints in the musculoskeletal system) volunteered (21 to 60 years) while both genders were divided into four age groups according to decades. A pressure measuring platform was used. Body sway and weight distribution were recorded in two dental occlusion conditions (a) in rest position and (b) symmetrical blocking (bicuspid region) by cotton rolls.

**Results:**

Both, the frontal sway and the sagittal sway reduced by 0.67 mm (t(724) = − 3.9 (*p* <  0.001)) and by 0.33 mm (t(724) = − 3.4 (p <  0.001)). The relative pressure under the left forefoot increased by 0.33% (t(724) = 2.88 (*p* <  0.001)) and the relative pressure overall under the forefoot increased by 0.67% (t(724) = − 3.4 (p <  0.001)). Gender-specific, age-specific and BMI-specific reactions could not be identified.

**Conclusions:**

Subjects, free of any TMD and with no complaints of the musculoskeletal system, show small changes of the body sway and weight distribution when biting symmetrically on a cotton roll. These changes are independent of age, gender or body mass index (BMI). Due to the relative large sample size, the presented results can also be seen as norm values when body sway is used as an additional assessment of a TMD.

## Introduction

To date, several reviews and meta-analyses [1–10] conclude that some connections exist between the temporomandibular joint (TMJ) and the body posture and body sway. For example, the review by Moon & Lee [7] confirmed that, theoretically, neurophysiological connections exist that allow an influence of the TMJ on postural stability. However, a cause-effect relationship is missing [3, 4].

During interdisciplinary treatments of patients, temporomandibular dysfunctions (TMDs) are sometimes assessed based on body sway or body positioning. Here, Perinetti et al. [1] questioned the clinical significance between temporomandibular disorders (TMD) and postural stability due to low diagnostic accuracy, the general low quality of the used study protocols or missing follow-up studies and the validity of posturography as a diagnostic test [6]. Regardless of the quality of posturography as a diagnostic test method, the posturography itself and the corresponding posturo-stabilometric parameters, such as postural sway and velocity of the center of pressure (CoP), showed acceptable reliability [5, 11, 12]. The repeatability of postural variables, like the sway, was confirmed by Ishizaki et al. [13], although it has to be considered that the postural stability can be influenced by the circadian rhythm [14]. Further, Perinetti et al. [5] concluded in their meta-analysis that a 25% difference of the sway variables is required to measure a true difference between various conditions.

This finding has to be verified in the present study. Since posturographic parameters are suitable for scientific studies but not yet in the clinical setting [3, 4], they will also be used in the present analysis.

It is the aim of the present study to analyse the difference of body sway and weight distribution between the dental rest position and the symmetrical occlusion blocking in the bicuspid area. From these results, we will define the normal variation within healthy subjects. This normal range of variation in the measurements will provide also an indication of the significance of the difference of these variables in a clinical setting. Only a deviation larger than this normal variation would be indicative of alterations to the original functionality of the TMJ.

Within the scope of the study design, since this investigation is part of a project [15], we recruited male and female subjects both free from signs of a TMD through their adult lifespan and free from subjective signs of acute or chronic complaints or injuries of the locomotor system. With a post hoc test the confounding factors age, gender and BMI were further analysed to observe any changes throughout the lifespan. The results will also help in defining clinically relevant changes of these variables, as we cover the range of changes in a healthy population. Therefore, the changes have to be larger than this range to be indicative of a non normal change in the clinical setting. Based on the literature [16] values, the size of the test group was calculated. The symmetric occlusal blockade was conducted with cotton rolls. The theoretical concept assumes that blocking the occlusion eliminates possible temporally and disruptive neurophysiological influences of the temporomandibular system (TMS) on caudally located body regions or structures [16–18]. For a true difference, not only a change of a measurement larger than the measurement error is required, but also a change that exceeds a physiological, healthy change.

Consequently, the following hypotheses will be investigated:

1. The body sway differs while bilaterally blocking the dental occlusion compared to a rest position of the jaw.
2. The weight distribution differs while bilaterally blocking the dental occlusion compared to the rest position of the jaw.
3. The difference in body sway between the two conditions is independent of age.
4. There is no gender difference of the weight distribution between the two dental conditions.

## Material and methods

### Subjects

Based on the study of Baldini et al. [19] the detectable change with the occlusion condition was in the range of a fifth of the standard deviation. A power analysis resulted in a minimal sample size of 324 (power = 0.8, 8 variables (Bonferroni correction for multi comparisons). For the goal of this study, to calculate the lower bounds for a true difference between rest position of the jaw and a blocked dental occlusion, we distributed the 324 subjects evenly over the age range from 20 to 60 in bins of ten years. In addition, we collected the same number of female and male subjects and therefore tried to measure more than 81 subjects in every decade in both the female and male cohort.

In this study, 725 (396f/329 m) subjects volunteered. Subjects have no acute TMD symptoms or acute or chronic dysfunctions of the movement apparatus at the time of measurement. Subjects ranged from 21 to 60 years in age for both genders (Table 1). Subjects were equally distributed over every year and a description of the individual decades is presented in Table 1.

**Table 1:** Biometric distribution of the investigated subjects

Sex	Age group	Age (Mean ± SD)	n	Height [m]	Weight [kg]	BMI [kg/m^2^]
female	21–60	39.69 ± 11.66	396	1.67 ± 0.06	66.46 ± 12.76	23.8 ± 4.6
male	21–60	41.16 ± 11.40	329	1.81 ± 0.07	85.38 ± 14.18	26.1 ± 3.7
female	21–30	25.03 ± 2.68	105	1.69 ± 0.06	60.32 ± 7.87	21.1 ± 2.6
female	31–40	35.12 ± 2.91	101	1.66 ± 0.06	66.82 ± 13.14	24.1 ± 4.6
female	41–50	45.15 ± 2.99	94	1.66 ± 0.07	70.15 ± 15.10	25.4 ± 5.3
female	51–60	55.18 ± 2.92	96	1.67 ± 0.06	69.20 ± 11.90	24.9 ± 4.4
male	21–30	25.19 ± 2.69	81	1.81 ± 0.07	77.66 ± 10.07	23.7 ± 2.4
male	31–40	35.82 ± 2.88	77	1.80 ± 0.07	86.30 ± 12.24	26.8 ± 3.6
male	41–50	55.18 ± 2.92	96	1.67 ± 0.06	69.20 ± 11.90	24.9 ± 4.4
male	51–60	55.08 ± 2.80	87	1.81 ± 0.08	88.50 ± 14.31	26.9 ± 3.6

According to the World Health Organization (WHO) classification [20], overall the women are of normal weight, while men are pre-obese. However, a detailed look into the age groups revealed, that women age 41–50 are pre-adipose (25.4 ± 5.3 [kg/m^2^]) and within the male cohort the age 31–40 and 51–60 are pre-adipose (26.8 ± 3.6 [kg/m^2^] and 26.9 ± 3.6 [kg/m^2^], respectively).

Before the study was conducted, each participant had to sign a written consent and complete a medical history form and anamnesis questionnaire (Centre for Dental, Oral and Maxillofacial Medicine of the Goethe University Frankfurt am Main [21]). The latter included questions on general diseases such as osteoporosis, diabetes mellitus, pain in the joints in general, noises in the ears as well as complaints in the temporomandibular joint. The test persons were also asked about possible accidents in the mouth, jaw and face areas and in the musculoskeletal system.

The study was in accordance with the 1964 Helsinki Declaration and its later amendments and was approved by the local medical ethics committee of the Faculty of Medical Science, Goethe University Frankfurt, Germany (approval No. 303/16).

### Measurement system

The pressure measuring platform GP MultiSens (GeBioM GmbH, Münster, Germany) has a measurement area of 38.5 × 38.5 cm, into which 2304 pressure sensors are integrated. The body sway and weight distribution can be measured with a sampling rate of 100 Hz. The sensors are arranged in a quadratic matrix and distributed at a density of 1.5 sensors/cm^2^. The maximum measurement error is ±5% (according to the manufacturer).

### Measure protocol

Each subject was instructed to stand within the circle depicted on the pressure plate. They should stand in habitual body position and fixing their sight on a point at eye level without moving. They were also asked to keep their heels on a predetermined line. The foot positioning was left to the test subject’s discretion. Two conditions were measured: rest position and cotton roll. In the rest position the jaw should be hold in a relaxed position. In the cotton role, condition subjects were instructed to bit with moderate biting force onto two cotton roles (left and right side). In every condition the body sway and weight distribution were recorded for 30 s. The recordings were repeated three times and the mean of body sway and weight distribution was calculated.

### Evaluation of parameters

The weight distribution of the left and right foot and the maximum body sway were recorded. The variables that were collected are: the amplitude of the frontal sway (mm), the amplitude of the sagittal sway (mm), percentage weight distribution of the four quarters relative pressure “left forefoot”, relative pressure “right forefoot”, relative pressure “left rear foot”, relative pressure “right rear foot”, the sum of the relative pressure “overall forefoot” and the sum of the relative pressure “left foot” (rear foot and right side were left out because they are the complementary to 100%).

### Statistics

Statistical analysis was done in matlab (Version 2018a). Significance was tested using a manova. Prior to the manova, data were inspected. Normal distribution was tested with the Lilliefor test [22]. In case of non-normal distribution, normal distribution was calculated through the rank transformation of the data. This was done for all variables. The difference of the rank distributed data was calculated and subjected to a manova. The Wilk test was used to evaluate the multiple comparisons. The response of the model were all 8 variables. No independent factors were tested. If significant, a post hoc student’s t-test was performed. To check the uniformity of the result over age and gender a second manova was done with the same response variables, however with the sex, age groups (as the decades) and BMI as the independent factors (Wilkinson notation: sex + age group + BMI + sex:age group + sex: BMI + BMI:age group). Again, as a post hoc test the student’s t-test or an anova was performed. For the post hoc tests a Bonferroni correction was applied. Data were presented as the original data (not rank transformed) for a better understanding. The 1st quartile, the median and the 3rd quartile were calculated and reported, to present a normal range of changes in healthy, symptom free people. Significance level was set to 0.05.

## Results

No variable was normally distributed. Therefore, all variables were transferred to a normal distribution via rank transformation. The manova test on the difference between rest position and cotton roll condition revealed a significant result for at least one variable F(7,718) = 8.14 (*p* <  0.001) (Table 2).

**Table 2:** Result of the manova. Differences could be observed within at least one variable over all subjects and between the sex group

Within	Between	Statistic	Value	F	R^2^	df1	df2	*P* value
variable	(Intercept)	Wilks	0.927	8.14	0.073	7	718	< 0.001

A student’s t-test showed that over all subjects the frontal and sagittal sway were reduced, and the overall forefoot and the left forefoot are increased for the cotton roll condition (Table 3). The frontal sway reduced by 0.67 mm (t(724) = − 3.9 (*p* <  0.001)) and the sagittal sway reduced by 0.33 mm (t(724) = − 3.4 (p <  0.001)). The relative pressure under the left forefoot increased by 0.33% (t(724) = 2.88 (p <  0.001)) and the relative pressure overall forefoot increased by 0.67% (t(724) = − 3.4 (p <  0.001)).

**Table 3:** Descriptive statistics of all the variables over the whole sample size. ^a^ behind the variable indicates a significant difference. For all variables the degree of freedom is df = 724

Variable names	Rest position			Cotton roll			Difference cotton roll – rest position			T -value	*P*-value
	Median	1st quantile	3rd quantile	Median	1st quantile	3rd quantile	Median	1st quantile	3rd quantile		
Body sway and weight distribution											
frontal sway [mm]^a^	11.33	8.67	15.54	10.67	8.00	14.33	−0.67	−3.00	1.33	−3,90	< 0,001
sagittal sway [mm]^a^	14.67	10.00	20.00	14.33	10.33	18.67	−0.33	−3.00	2.33	−3,36	< 0,001
left forefoot [%]^a^	19.33	15.33	23.33	19.67	15.33	24.33	0.33	−1.67	2.00	2,88	< 0,001
right forefoot [%]	15.00	11.00	19.33	15.00	11.00	19.67	0.00	−1.67	1.67	0,49	0,62
left rearfoot [%]	32.33	27.67	36.67	31.67	27.33	37.00	0.00	−2.33	2.08	−1,57	0,12
right rearfoot [%]	32.67	27.67	38.00	32.50	27.00	38.00	0.00	−2.67	2.33	−1,18	0,24
left foot [%]	51.89	47.33	57.00	52.33	47.58	57.00	0.00	−2.00	2.33	1,46	0,14
overall forefoot [%]^a^	33.33	28.33	41.00	34.33	28.33	42.00	0.67	−2.00	3.33	3,38	< 0,001

The second manova checked the differences between sex, age decades or BMI revealed no difference between either sex, age decade, BMI or any interaction of sex and age decade, sex and BMI or BMI and age decade (Table 4). The lowest probability for a difference was found between the interaction between sex and age decades by *p* = 0.54.

**Table 4:** Result of the manova. No difference could be observed between sex, age decades or BMI or between the interactions between any of the three combinations

Within	Between	Statistic	Value	F	R^2^	df1	df2	*P* value
variable	sex	Wilks	0.99	0.50	0.00	7	712	0.83
variable	Age decade	Wilks	0.99	0.841.04	0.01	7	712	0.56
variable	BMI	Wilks	1.00	0.29	0.29	7	712	0.96
Variable	sex:age decade	Wilks	0.99	0.86	0.01	7	712	0.54
Variable	Sex:BMI	Wilks	0.99	0.84	0.01	7	712	0.55
variable	BMI:age decade	Wilks	1.00	0.32	0.00	7	712	0.95

## Discussion

The blocked occlusion condition introduces small but significant decreases of the frontal (0.67 mm) and sagittal sway (0.33 mm). Therefore, the 1st hypothesis can be accepted. In addition, the pressure distribution moves slightly to the front with the blocked occlusion condition. The relative pressure under both forefeet increase by 0.67%, where the dominant leg seems to be the left forefoot with an increase of 0.33%. Only two of six pressure variables showed an effect in the cotton roll occlusion condition. Therefore, the 2nd hypothesis has to be rejected.

To begin with the tested population, free of any TMD and with no complains of the musculoskeletal system, had small changes of the pressure distribution with the blocked dental occlusion condition.

Based on the presented results, the change between rest position and blocked occlusion is independent of differences between gender, age or BMI. Therefore, the 3rd and 4th hypothesis can be accepted. It has been reported that male subjects have a larger postural sway than female subjects, [23–25] and that the postural stability does decrease with age [26, 27]. However, this did not affect the influence of the occlusion conditions’ change. Additionally, we have found no difference between any age and gender group for the change of the pressure variables measured. The body mass and body mass index (BMI) of the cohort has been compared with the general German distribution [28, 29]. Only marginal differences in any age or gender group could be found. Even though weight might be another cofactor, the results, in terms of a true difference, match the population and are therefore representative.

The differences reported in this study are for healthy subjects, free of any TMD and with no complaints of the musculoskeletal system. If body sway is used as an additional parameter to assess a TMD, the introduced change has to be larger than the difference reported here, for a possible indication of a TMD. Therefore, this might be seen as the minimal deviation to be a true difference in terms of being outside the norm range. For a possible relevant outcome, the observed changes have to be outside the range of the 1st to 3rd quartile (Table 3). For instance, the frontal sway has to be smaller than 3 mm or larger than 1.33 mm (sagittal sway < 3 mm; > 2.33 mm) with the blocked occlusion condition, compared to rest position, to be a true difference. This is also true for the relative pressure of the forefoot being smaller than 2% and larger than 3.33%, and the left foot being smaller than 2% and larger than 2.33%. The minimal changes shown in Table 3 can be used for both sex and any age decade or BMI, as no gender, age or BMI specific differences were measured.

From this study we cannot claim that a difference larger than the measured difference is due to a TMD. On the one hand, TMD was not addressed within this study. On the other hand, body sway and pressure distribution are the result of complex control functions of the body, and therefore dependent on many more factors.

Many researchers calculated effects of occlusion conditions on various parameters of the pressure distribution [2, 19, 30–32]. These previous analyses have focused primarily on whether changes in the TMJ exist in principle, which has been confirmed in the subsequent studies: Bracco et al. [30] measured a reduction in body sway with a myocentric jaw position that is comparable with the values presented in this study. The influence of an occlusion condition is by far smaller than other interventions, such as closed eyes, according to Baldini [19]. Contrary to our results, Amaricai et al. [31] could not observe any change of the weight distribution between the rest position and any symmetrical occlusion condition. Also, a recent study by Michalakis et al. [32] measured significant changes in the lateral direction (sagittal sway) but no changes in the anterior posterior direction. The study involved 20 subjects (14 m/6f), and they counted the number of subjects that showed a change.

Several explanations are discussed in the scientific literature to give a theoretical explanation of the observed phenomena. The most common explanations can be categorized into two categories: sensory dependent theory [7, 33] and mechanical dependent theory [34, 35]. The sensory dependent theory states that, missing sensory information in the occlusion condition alters brain circuits that subsequently lead to a change of the motor pathways [7, 33, 36, 37]. The mechanic dependent theory states that either a relaxation of muscles of the anterior triangle or a change of the air tunnel lead to a repositioning of the head, and therefore leads to a change of body positioning and subsequently to a change of the pressure distribution [34, 35, 38, 39]. Unfortunately, to date none of the theories has been investigated more deeply.

There are some limitations of this study. The inclusion criteria are subjects free of TMD symptoms. For this assessment a standardized questionnaire was used [21]. Qualified orthodontists/dentists evaluated this questionnaire and discussed open questions in an interview. No excessive dental/orthodontic or orthopaedic examination was undertaken. Furthermore, only a symmetrical occlusal blockade and not a unilateral blockade was purposely chosen. This was to avoid that a unilateral blockade would simultaneously cause a compression in the ipsilateral temporomandibular joint including the corresponding neurophysiological reactions. Our intention was only the bilateral exclusion of function and muscle retractions in the TMS.

In this study, only the correlation between the change of the occlusion condition (between rest position and symmetrical blocked occlusion) and spatial pressure parameters were calculated. This is obviously no investigation of a cause and effect relationship, nor does this study address the theoretical background. Several research reviews and meta-analysis have shown that a change of the occlusion condition can affect the remaining body, however a deeper understanding of the underlying mechanism is required to optimise the interventions [1–7]. For instance, a time dependent analysis of the COP path might be useful to determine if the control strategy, and therefore the sensory pathway, is effected with a change of the occlusion condition [40].

This study can be used as a baseline to help classify future measured changes in body sway and weight distribution (age or gender specific), as to assess whether they are normal variations or indicative of a true difference. Furthermore, the clinicians can use the norm values to compare their findings with the findings in healthy, TMD free and without any complains in the musculoskeletal system. Thus, a natural variance can be differentiated from a pathological one, which in the case of the latter, should be followed by a therapeutic consequence.

## Conclusion

Subjects, free of any TMD and with no complains of the musculoskeletal system, show small changes of the body sway and weight distribution when biting symmetrically on a cotton roll. These changes are independent of age, gender or BMI. Due to the relatively large sample size, the presented results can be seen as norm values when body sway is used as an additional assessment of a TMD.

## Data Availability

All data generated or analysed during this study are included in this published article.
